# 

**Published:** 1808-12

**Authors:** 


### NEW MEDICAL PUBLICATIONS.

Modern Medicine, containing a brief Exposition of the Discoveries and Doctrines that have occasioned the recent Advancement of Medical Practice, and an Inquiry how far the Principles of the Healing Art may become the Subjects of unprofessional Research. By David Uwins, M.D. 8vo. 6s.

An Answer to Mr. Highmore's Objections to the Bill before Parliament, to prevent the spreading of the Infection of the Small-pox. By Charles Murray.

A Review of the Report of the Royal College of Physicians of London on Vaccination. By Benjamin Mosely, M.D. 3s.

Identities Ascertained, or an Illustration of Mr. Ware's Opinion respecting the Sameness of Infection in Venereal Gonorrhœa and the Ophthalmia of Egypt. 8vo. 2s. 6d.

---

### To CORRESPONDENTS.

We are much obliged to C. B. for the polite compliment with which he honours one of us, but regret that such a passage prevents our transcribing his letter. We assure him that he mistakes us, if he conceives we venture to alter communications with the names of the author annexed. But he is not less mistaken if he expects our Journal to become a vehicle for *mirth*, especially at the expence of those ingenuous writers, who, in the midst of a most laborious employment, are as willing to give as anxious to receive, every information in a science which can never be perfect. The punishment which the wisest man allots to those "who are wise in their own conceit," is, we doubt not, a very proper one, and should often be perused by us all. We recollect that the same wise man remarks, The beginning of strife is like the letting out of water.

We thank Mr. Davis for his very candid communication, which shall be inserted in our next, if he will permit us to shorten it; or if he will favour us with his address, we will return it to him for that purpose.

Mr. Roberton's paper shall appear in our next.

Press of matter on temporary subjects, obliges us to defer Dr. Ferguson's paper.

Medicus's Letter cannot be admitted in its present form. If he will send us a proper address, it will be returned with such remarks as will render it admisable.

Dr. Arnold and Jacobus came too late for insertion, but shall appear in our next.

---

### CORRIGENDA.

Line 16, page 499, after the word growth, read, "intimately connected with the principle of irritability is the power of evolving heat."

Line ditto, page ditto, for this property, which is peculiar, read, "this property, which is *also* peculiar.

Line 11 from the bottom, page 544, for *never*, read *scarcely ever*.

---

END OF VOL. XX.

### INDEX.

ABSTINENCE, fingular cafe of 402
———————, further account of ditto 527
Acid formed in indigeftion, on the 193
——, internal ufe of arfenic 494
Action, on affociated 111
Acute organic affections, on blood-letting in 536
Adams, Dr. on vaccination 26
——— Dr. on blood-letting 536
——— on opium 567
Addrefs to the readers of the Medical Journal 387
——— to the faculty 253
Adhefive ftraps in ulcers, on 110
Affections, on inflammatory 78
———, cafe of fingular nervous 224
Affufion, on cold 51, 81, 164
Agaricus, defcription of the 352
——— glutinofus, account of 566
Age, on certain effects of 154
Ague, on the prevalence of the 479
Air, fire produced by the compreffion of atmofpheric 287
Alkalies, on the decompofition of the 95
Ammonia, experiments on 96
Anatomy, divifions of 43
Aneurifm, cafe of 264
——— cafe of popliteal 480
Anguftura bark, obfervations on 21
Animation, cafes of fufpended 236
Aphthæ, on the treatment of 94
Areola, on the vaccine 337
Armftrong's, Mr. cafe of hydrophobia 323, 520
Arum cordifolium, ufes of the 499
Afcites, on the treatment of 262
Afthma, obfervations on 342
———, account of a fpecies of 554
Atkins's, Mr. hydrometer defcribed 48
Atmofphere, on the action of the 5
———, natural hiftory of the 365
Authors on the venereal difeafe 560

Badham, Dr. on inflammatory affections 78
Baltimore, Medical College founded at 385
Bandage, on the paper and plafter 255
Barracuda, on the poifon of the 463
Bartholomew's hofpital, lectures at 288
Bed for the fick, defcription of a 142
Beddoes, Dr. his cafes of hydrophobia 195
Beddoes, Dr. cafes and diffections 407, 540
Bell's, Mr. treatife on tumours, remarks on 242
Bellot, Mr. in reply to Mr. Bradley 259
——— anfwer to 508
Blair's, Mr. explanatory letter 60
Blane, Dr. on yellow fever 31
Blood-letting, obfervations on 391, 536
Boletus, defcription of 353
Bones, on the ufe of medulla in 286
BOOKS, CRITICAL ANALYSIS OF NEW MEDICAL — Obfervations on the Inflammatory Affections of the Bronchiæ, by C. Badham, M. D. 78. Expofition of the Practice of affufing cold Water as a Cure for Fever, by R. Jackfon, M. D. 81, 164. The Edinburgh Medical and Surgical Journal, 171, 461. Effays on the Morbid Anatomy of the Human Eye, by J. Wardrop, 182. Defcription and Treatment of Cutaneous Difeafes, by R. Willan, M. D. 273, 371. Reports on Vaccination, with Remarks and other Papers, by J. Adams, M. D. 282. A Letter on Vaccination, by T. Wadley, *ib.* Vaccination Vindicated, by James Rumfey, *ib.* A General View of the Natural Hiftory of the Atmofphere, by H. Robertfon, M. D. 365. Cafes of Diabetes, Confumption, &c. by Robert Watt, 547. Letter to the Right Hon. Spencer Perceval, on Regulating the Practice of Variolous Inoculation, by Sir E. Carrington, 557. Important Refearches upon the Venereal Affection in Pregnant Women, &c. by Dr. Mahon, tranflated by Jeffe Foot, 560
BOOKS, NEW, ANNOUNCED.—Sonini's Work on Birds, 98. Dr. Alley on Hydrargyri, *ib.* Dr. Reid's Reports of Difeafes, *ib.* Mr. Watt on Diabetes, 194. Dr. Davis on Carditis, 290. Mr. Watt's Anatomico-Chirurgical Veins of the Nofe, &c. 386. Dr. Grant's Hiftory of Brazil, *ib.* Dr. Smith's Botanical Illuftrations, *ib.* Mr. Carmichael's Effay on Carbonate of Iron in Cancer, 482. Dr. Lambe's Reports on Cancerous Tumours and Ulcers, 569. Mr. Sylvefter's Treatife on Chemiftry, *ib.* Mr. Taunton's Treatife on Pathology, *ib.*
Boftock, Dr. reply to 181

Botanical defcription of Britifh plants 157, 267, 349
——— ftudies, on the utility of 14
Bougie, defcription of a new 311
Bourne, Dr. on a cafe of abftinence 527
Bradley, Mr. on crural hernia 508
———, Dr. on hydrophobia 547
Bronchial polypi, cafes of 471
Brookes, Mr. his models defcribed 3
Burns, on the treatment of 56, 490
Bufh, Mr. on hernia umbilicalis 31
Cadiz, account of yellow fever at 34
Cambridge, fuppofed failures of vaccination at 363
Cancer, effects of opium in 567
Canine madnefs, remarks on 9
———, remedies for 10, 271
Cantharides, on the internal ufe of 291, 429
Carrington, Sir E. his letter to Mr. Perceval 557
Carter, Mr. on rheumatifm 246
Carwardine's, Mr. cafe of tumour 483
Cafes and diffections 540
Cataract, on extracting the 174
Caterpillars, mode of expelling 384
Celtis auftralis, on the 9
Champignons, on the poifon of 54[illegible]
Chatard's, Dr. cafe of fiftula lachrymalis 147
Cheltenham, analyfis of the waters of 193
Cheyne, Dr. on purgative medicines 180
——— his cafe of bronchial polypi 471
Childbearing, on leffening the pains and danger of 73
China, ftate of vaccination in 483
Chifholm, Dr. on the poifon of fifh 461
Cholera, cafes of 382
Chorea 555
Climate, on the effects of 240
Cochineal infect, a reward offered for a live 480
Coffee, analyfis of 384
Colchefter medical fociety, meeting of the 565
Colica faturnina, on 342
Confumption, cafes of 547
Contagion, obfervations on 34
Convulfions, on puerperal 492
Cordilleras, account of the 11
Corn, on the faccharine matter in Indian 482
Cornea, on the mortification of the 174
——— on the fenfibility of the inflamed 472
Cow-pock failure, cafes of 61, 283
Cow-pox, remarkable cafe of 535
Crichton's, Col. bed for the fick defcribed 142
Crocker, Eliz. on the cafe of 251
Cromftock's, Dr. cafe of nervous affection 224
Crural hernia, how to reduce 4
——— obfervations on a cafe of 63, 508
Curbinata, defcription of the 287
Davy's Mr. experiments on the alkalies 95
——— on the decompofition of the earth 383
——— galvanic apparatus, defcription of 254
Diabetes, cafes of 547
Diarrhœa, obfervations on 379
Dickfon, Dr. on yellow fever 473
Digeftion, obfervations on 2
Digitalis, on the action of 13
———, on the ufes of 328, 523
Difeafes in London, monthly report of 94, 192, 284, 382, 479, 564
——— in Edinburgh, reports of 89, 190, 283, 378, 477, 561
Diffections, account of 401
Dogs, on the madnefs of 9
Dorr, Mr. on injury of the fpine 111
Drugs, on the fophiftication of 20
Duck, Mr. on the cafe of E. Crocker 254
Dunn, Mr. on the ufe of arfenic acid 494
Earths, on the decompofition of the 383
Edinburgh, report of difeafes at 89, 190, 283, 378, 477, 561
——— Medical Journal, review of the 171, 461
Edwards, Mr. on umbilical hernia 405
Elba, new mineral found in the ifle of 97
Electrical apparatus, conftruction of a new 287
Ellis, Mr. on refpiration 181
Endemic cynanche parotidea, inftance of 180
Enometer, a new inftrument fo called 482
Epidemic, account of an 392
Eryfipelas, on 371, 342
Erythema, obfervations on the 281
Ether, on the muriatic 125
Etymology, remarks on 535
Experiments on the alkalies 95
——— on the friction of fluids 97
——— on refpiration 98
——— on muriatic ether 125
——— on mineral waters 133
——— on living animals 174
——— on the acid formed in digeftion 193
——— on the waters of Cheltenham *ib.*
——— to difcover the ufe of the fpleen 215
——— electrical 287
——— on iron 347
——— on the decompofition of the earths 383
——— galvanic 475
——— on Indian corn 482

Eye, on the morbid anatomy of the 182
Fern, defcription and ufes of the male 158
Fever, on Dr. Clutterbuck's theory of 25
——, on cold affufion in 51, 81, 164
Fire produced by the compreffion of atmofpheric air 287
Fifh, defcription of a remarkable 287
—— on the poifon of 461
Fiftula lachrymalis, cafe of 147
Flax, method of preferving it from caterpillars 384
Fleet, falutary improvement in the Englifh 37
Fluids, on the friction of 47
Foot, Dr. on the functions of the liver 339
Framboefia, cafe of 401
Fuci, defcription and ufes of the 349
Gall, Dr. on the fyftem of 17
Galvanic battery, defcription of a 254
Galvanifm, obfervations on 474
Generative organs, difeafes in the 429
George's St. hofpital, lectures at 385
Glafgow, medical lectures at 288
Goodhall, Mr. on vaccination 333
Gout, on the treatment of 65
Groom, Mr. on fufpended animation 235
Guy, Mr. on afcites 262
Guy's hofpital, lectures at 288
Hall, Dr. on a cafe of yaws 401
—— on irritability of vegetables 499
Hardy, Mr. on cow-pock failure 237
Harleftonenfis, controverfy with 134, 145, 266, 328
Harriott, Mr. on cold affufion 52
Hemorrhagic difpofition, account of an 69
Hernia, method of reducing crural 4
—— remarks on a cafe of 63
—— umbilicalis, on the treatment of 312
—— obfervations on 405
—— on a cafe of crural 508
Hiftoric fketch of the progrefs of medicine 1
Hodgfon's Mr. cafe of rabies 531
Home, Mr. on diffection 2
—— on the ftructure and ufes of the fpleen 211
Horizon, defcription of an artificial 287
Hunter, John, on Venereal affections 561
Hydrometer, defcription of a new 481
Hydrophobia, obfervations on 8
—— Dr. Beddoes's cafe of 195
—— Mr. Smith's cafe of 198
—— Dr. Powell's cafe of 201
—— Dr. Walker, on 209
—— remedies for 271
—— Mr. Armftrong, on 323, 520
—— Mr. Loudhouter's cafe of 326
—— on preventing death in 358
—— Mr. Thomfon's cafe of 396
Hydrophobia, remarks on cafes of 451
——, Mr. Hodgfon, on 531
——, Dr. Bradley, on 543
Hyfteria, obfervations on 7
Iceland, lichen, defcription and ufes of the 268
India, ftate of vaccination in 25
Indian corn, experiments on 482
Indigeftion, on the acid formed by 193
Infantile difeafe, hiftory of a cafe of 57
—— obfervations on 237
Infants, on venereal affection in 560
Inflammation, on the nature of 174
Inflammatory affections, on 78
Inoculation, obfervations on 258
Intelligence, mifcellaneous 95, 193, 286, 383, 479, 565
Ipecacuanha in fever, on 52
Iron, on the converfion of it into fteel 347
—— on the ufe of carbonate of 447
Ifchuria, fingular cafe of 115
Jackfon, Dr. on cold affufion in fever 81, 164
Jackfonian prize for 1809 286
Jenite, a new mineral fo called 97
Jennerian fociety, report of the royal 363
Kentifh, Dr. obfervations on his controverfy 53
—— his reply to Dr. Wood 56
Key's Mr. cafes of cow-pock failure 61
Kinglake, Dr. on the treatment of gout 65
—— on blood-letting 391
Lamb's Mr. method of making fea water frefh 193
Lawrence, Mr. on a peculiar affection of the teftis 171
LECTURES, MEDICAL ANNOUNCED.— Dr. Clough, on Puerperal Anatomy, Phyfiology, and Pathology, 194, 290. In the Univerfity of Glafgow, 288. At the London Hofpital, *ib.* St. Bartholomew's Hofpital, *ib.* At St. Thomas's and Guy's Hofpitals, *ib.* Mr. Brookes, on Anatomy, Phyfiology, and Surgery, 289. Mr Taunton, on Anatomy, Phyfiology, Pathology, and Surgery, *ib.* Mr. Thomas, on the Principles and Operations of Surgery, *ib.* Mr. Wilfon, on Anatomy, Phyfiology, Pathology, and Surgery, *ib.* Mr. Accum, on Experimental Chemiftry and Analytical Mineralogy, *ib.* Dr. Buxton, on the Theory and Practice of Medicine and the Materia Medica, *ib.* Mr. Carpue, on Anatomy, Surgery, &c. *ib.* Dr. and Mr. Clarke, on Midwifery, &c. *ib.* Dr. Clutterbuck, on the Theory and Practice of Phyfic, &c. 290, 386. Dr. Dennifon, on Midwifery, &c. *ib.* Mr. Moor, on the Teeth, *ib.* Dr. Pearfon, on Phyfic and Chemiftry, 385. Mr. Chevalie on the Principles and Operations of Surgery, *ib.* Mr. Carlifle, on Surgery, 386. Dr. Reid, on the Theory and Practice of Medicine, 482.
Leg's Mr. invention for clearing water from ponds 383
Leyden phial, improvement in the 287
Lichen iflandicus, on the 332
Lichens, defcription and ufes of the 267
Light, fenfibility of inflamed cornea to the 472
Liver, on the functions of the 339
London, reports of difeafes in 94, 192, 285, 381, 479, 564
——— remarkable heat in 6
——— hofpital, lectures at the 288
——— pharmacopœia, account of the new 137
——— vaccine inftitution, to the correfpondents of the 250
Loudhouter, Mr. on hydrophobia 326
Lungs, inftance of condenfed 178
Madnefs, obfervations on canine 9, 468, 520, 543
Mahon, Dr. on venereal affection 560
Mammoth, difcovery of a complete 18
Marchantia, defcription and ufes of the 159
Martin, Mr. on cold affufion in fever 51
Maryland, medical inftitution in 385
Maton's Dr. communication on vaccination 488
Maunoir, M. on extracting the cataract 174
Medical fcience, tables of the natural fyftem of 39
——— and Phyfical Journal, addrefs to the readers of the 387
Medicine, hiftorical account of the progrefs of 1
Medulla of bones, ufes of 286
Metals, experiments on new 95
Miller, Dr. on yellow-fever 28
Mineral, defcription of a new 97
——— waters, on fulphureous 133
——— acid gafes, ufes of the 35
Morbid anatomy, on the term 535
Morrifon's Mr. cafe of purulent ophthalmia 57
——— obfervations on ditto 237
Mofsman, Dr. on digitalis 328
Muriatic ether, memoir on 125
Mufhrooms, on poifonous 567
Myrrh mixture, on the 332
Navy, great improvements in the 37
Nervous difeafes, obfervations on 7
——— affection, fingular cafe of 224
New-York, defcription of 29
Noble's Mr. account of an endemic 180
Norris, Mr. on the death of Mr. Porfon 389
Nottingham, medical reports for 467
Novels, danger of reading 8
Oenanthe crocata, poifonous 15
Oil extracted from fun-flowers 385
Ophthalmia, on the Egyptian 11
——— cafe of purulent 57
——— defcription of 90
——— obfervations on 237
——— treatment of 190
Opium, on the folution of 141
——— effects of in cancer 567
Organic affections, on blood-letting in acute 536
Otto's, Dr. account of an hemorrhagic difpofition 69
Oxyd of bifmuth, on the 332
Paper bandage, on the 236
Parker's, Dr. cafe of ifchuria 115
Parrott, Mr. on the poifon of champignons 457
Parry's, Dr. method of preferving wood 384
Pafcalis, Dr. on fcarlatina anginofa 121
Pearfon, Mr. on vaccination in China 488
Pemphigus, on the 376
Perperes, Mr. on indigeftion 193
Pharmacopœia, new edition of the London 137
Phlegmona of the extremities, on 343
Phoenix, Mr. on electricity 287
Phthifis, efficacy of digitalis in 13
Phyfiology, lecture on 99
Phyficians, on the report of the royal college of 282
Pinkard, Dr. on the effects of climate 240
Plague, ftatute on the 558
Plants, obfervations on poifonous 15
——— defcription of Britifh 157, 267, 349
Plafter bandage, on the 235
Plethora, cafes of 556
Pneunomia, on the treatment of 191, 244
Poifon of champignons, fatal cafe of 457
——— of fifh, on the 461
Polypody, defcription and ufes of the 157
Pomphylox, varieties of 378
Potfon, Mr. account of the death of 389
Potaffium, a new metal defcribed 95
Potatoes, on the fecula of 568
Powell's, Dr. cafe of hydrophobia 201
Prieftley, Dr. on the converfion of iron into fteel 347
Prunus lauro ceratus defcribed 15
Publications, new medical 98, 194, 386, 482, 570
Puerperal convulfions, on 492
Purgative medicines, on the effects of 180
Purpura, defcription of the 280
Purulent ophthalmia, cafe of 57
——— obfervations, on 237
Purton, Mr. on the treatment of burns and fcalds 490
Quarantines, on 559
Quinquina, new fpecies of 18

Rabies, obfervations and cafes of 271, 469, 481, 531, 543
Reade, Dr. on the friction of fluids 97
Remedies, enquiry after popular 253
Refpiration, experiments on 98
———, Mr. Ellis's obfervations on 181
Rheumatifm, on chronic and acute 246
——— obfervations on 356
Ring, Mr. on the reports concerning vaccination at Cambridge 563
Roberton's, Mr. reports of difeafes 89, 190, 283, 378, 477, 561
——— on cures by the internal ufe of cantharides 291, 429
Robertfon, Dr. on the natural hiftory of the atmofphere 365
Rofeola, defcription of 278
Royfton's. Mr. fketch of the progrefs of medicine 1
Rubeola, obfervations on 467
Rumfey, Mr. on vaccination 282
Ruptured poor, new fociety for the relief of 193
Rufh, Dr. on leffening the pains of child-bearing 73
——— on preventing death in hydrophobia 358
Scalds, on the treatment of 54, 491
Scarlatina cynanchina, obfervations on 121
——— prevalence of 478
Scriblerus, on etymology 535
Scrophulous fores cured by cantharides 291
——— fwellings, remedy for 349
Sea, treatment of ulcers at 322
—— water, method of making it frefh 193
Shoolbred, Mr. on vaccination in Bengal 23
Sick, defcription of a bed for conveying the 142
Simmon's, Mr. on the plafter and paper bandage 255
Singer's, Mr. improved electrical apparatus 287
Skin, on difeafes of the, 273, 371, 473
Skrimfhire, Mr. on the fecula of potatoes 568
Small-pox, on inoculation for the 259
Smellie, Mr. on the treatment of ulcers 322
Smith's, Mr. cafe of hydrophobia 198
Sodaium, a new metal defcribed 95
Sphacelation, on 344
Spine, cafe of injury done to the 111
Spleen, on the ftructure and ufes of the 211
Staines, Mr. on inoculation 258
Steel, on the converfion of iron into 347
Storm, a remarkable 384
Straps in ulcers, on adhefive 110
Strictures of the urethra, on 314, 429
Strowger, Mr. in anfwer to Harleftonenfis 145
Strowger, Mr. reply to 266
——— vindicated 333
Sun-flower, ufe of the oil of 385
Sulphureous mineral waters, obfervations on 133
Surgeons, college of, their prize for 1809, 286
Surgery, on the art of 3
Sufpended animation, cafes of 235

Tables of medical fcience 50
Talleyrand, fpecimen of his writing 251
Tape worm, remedy for the 158
Tarantula, cafe of the fuppofed bite of 224
Taylor, Mr. his cafe of abftinence 402
——— on digitalis 523
Tellegen, Dr. on vaccination 334
Teftis, on a peculiar affection of the 171
Tetanus, German method of treating 8
———, cafe of 285
Thenard, Mr. on the muriatic ether 125
Thomfon's Mr. cafe of hydrophobia 396
Tic douloureux, cafe of 180
Time, on our perceptions of 154
Tokay, a great ftorm in 384
Trees, efficacious method of propagating fruit 569
Tremella, defcription and ufes of 272
Tumour, cafe of 483
———, on Mr. Bell's treatife on 242
Typhus icteroides, on 27
——— fever, prevalence of 478
Ulcers, on adhefive ftraps in 110
——— on the treatment of 322
Umbilical hernia, remarks on 405
Urethra, on ftrictures of the 314
———, on affections of the 436
Urticaria, defcription of the 273
Uva urfi, on the 332
Vaccination, ftate of 21
——— Mr. Key, on 61
——— Mr. Hardy, on 237
——— Dr. Adams, on 282
——— Mr. Wadley, on *ib.*
——— Mr. Goodhall, on 333
——— Dr. Tellegen, on 335
——— on a fuppofed failure of 363
——— in China 488
——— Sir E. Carrington, on 557
——— refolutions of the Colchefter fociety, on 565
Vaccine areola, on the 337
——— pock, on the 534
Vegetables, on the irritability of 499
Venereal affection in pregnant women, on 560
Verax, reply to 65
Vetch, Dr. on ophthalmia 10
——— on the fenfibility of the inflamed cornea 472
Wadley, Mr. on vaccination 282
Walker, Mr. on medical fcience 39
——— his lecture on phyfiology 99

Walker, Mr. on the medulla of bones 286
———, Dr. on the conduct of 60
——— on our perceptions of time 154
——— on hydrophobia 209
——— to his correfpondents 250
——— on the vaccine areola 337
Wardrop, Mr. on the human eye 182
Warnock, Mr. on adhefive ftraps in ulcers 110
Waters, on fulphureous mineral 133
——— method of clearing fuperfluous 383
Watt's, Mr. cafes of diabetes 547
Weather, ftate of the 561
Weftrumb, Mr. on mineral waters 133
White's Dr. cafe of aneurifm 264
Willan, Dr. on cutaneous difeafes 273 371
Wilfon, Dr. on fever 36, 174
Women, on the venereal affection in pregnant 560
Wood, Dr. on the controverfy between him and Dr. Kentifh 53
——— reply to 56
——— caufes of the decay of 384
Wright, Mr. account of his portable horizon 287
Writing, on bad 250
Yaws, on a cafe of 401
Yellow fever, Dr. Miller on 27
———, Dr. Dickfon on 473
Zizania aquatica, propagation of the 19

### REFERENCES to the PLATES.

| | PAGE |
|---|---|
| Professor Davy's Galvanic Apparatus — — | 254 |
| Poisonous Fungi, and genuine Champignons — | 457 |
| Mr. Carwardine's Case of Tumour — — | 483 |

